# The impact of tris(pentafluorophenyl)borane hole transport layer doping on interfacial charge extraction and recombination

**DOI:** 10.3762/bjnano.16.52

**Published:** 2025-05-21

**Authors:** Konstantinos Bidinakis, Stefan A L Weber

**Affiliations:** 1 Max Planck Institute for Polymer Research, Ackermannweg 10, 55128 Mainz, Germany; 2 Institute for Photovoltaics, University of Stuttgart, Pfaffenwaldring 47, 70569 Stuttgart, Germany

**Keywords:** cross-section, hole transport layer doping, Kelvin probe force microscopy, perovskite solar cells

## Abstract

Selective charge transport layers have a strong influence on the overall efficiency and stability in perovskite solar cell devices. Specifically, the charge extraction and recombination occurring at the interfaces between the perovskite and these materials can be a limiting factor for performance. A lot of effort has been put into improving the conductivity of selective contacts, as well as the junction quality and energetic alignment with the absorber. On the hole extracting side, organic semiconductors have been extensively used due to their flexibility and favorable properties. Two of such compatible materials that have yielded high performing devices are the small molecule 2,2',7,7'-tetrakis[*N*,*N*-di(4-methoxyphenyl)amino]-9,9'-spirobifluorene (spiro-OMeTAD) and the polymer poly[bis(4-phenyl)(2,4,6-trimethylphenyl)amine] (PTAA). In this work, we investigate the impact of hole transport layer doping on the performance and potential distribution in solar cells based on these materials. To do so on operating solar cells, we created samples with exposed cross-sections and examined their potential profile distributions with Kelvin probe force microscopy (KPFM), implementing our comprehensive measurement protocol. Using the Lewis acid tris(pentafluorophenyl)borane (BCF), we enhanced the hole extracting material/perovskite junction quality in spiro-OMeTAD and in PTAA based devices. Measurements under illumination show that the improvement is caused by a reduced recombination rate at the perovskite/hole transporter interface.

## Introduction

Perovskite solar cells (PSCs) are a promising class of photovoltaic material that exhibits high power conversion efficiencies and relies on a low-cost solution-processed fabrication method [1–4]. At the core of their success lies the perovskite absorber material, which exhibits impressive bulk properties, such as long carrier lifetimes and low recombination rates [5–8]. However, the granular nature of perovskites and the layered structure of their solar cells, introduce complications such as grain boundaries and interfacial defect states that hinder performance. Specifically, since the interaction of adjacent layers at the interfaces of a solar cell is an important limiting factor for its operation, there is a need for dedicated studies regarding interfacial behavior. Kelvin probe force microscopy (KPFM) is an important tool for conducting such studies, enabling the measurement of the perovskite’s surface potential by monitoring the electrostatic force between the surface and a conductive probe (See Supporting Information File 1, Section 1). This measurement can provide insights about charge generation and transport within the absorber material, as well as charge extraction to the relevant interfaces [9–12].

The details of interfacial electronic carrier extraction at the junctions of the perovskite with the electron and hole transport layers (ETL, HTL) define the ability of a solar cell to generate electrical current effectively. Particularly, the relative capability of the two interfaces to properly extract and block charges is critical, since issues such as energetic misalignment, trap states, and interfacial recombination may lead to an uneven extraction and therefore a charge accumulation within the perovskite. Initial studies suggested that this asymmetrical charge carrier behavior indicates an unfavorable hole extraction and a promoted electron extraction [9,13–14], but the migration and interaction of mobile ions (such as I^−^ ions interacting with 2,2',7,7'-tetrakis[*N*,*N*-di(4-methoxyphenyl)amino]-9,9'-spirobifluorene (spiro-OMeTAD) [15–16] and Li^+^ ions interacting with TiO_2_ [17–18]) has also been proposed to explain the asymmetrical distribution of charges within the perovskite [19–20].

Many research endeavors involve the optimization of ETLs in terms of passivation, post-fabrication treatment, and choice of optimal materials [21–23], leaving research on HTL optimization vastly overlooked. In regular n-i-p architecture devices mostly two organic semiconductors have been used as HTL in the past: spiro-OMeTAD and poly[bis(4-phenyl)(2,4,6-trimethylphenyl)amine] (PTAA) [24]. These compounds exhibit favorable solubility, reasonable energetic alignment with most perovskites, and an amorphous nature. The main issues that arise from their usage involve poor conductivity and mechanical stability [25], the existence of pinholes, and a poor adhesion with the adjacent perovskite. There have been many studies trying to address these points and advance PSC performance through HTL optimization, with conventional approaches mainly focusing on the doping strategies applied to these two materials [26–29].

The organic semiconductors spiro-OMeTAD and PTAA are traditionally doped with the ionic p-dopant bis(trifluoromethane)sulfonimide lithium salt (LiTFSI) and 4-tertbutylpyridine (tBP). In the case of spiro-OMeTAD, in presence of oxygen, LiTFSI promotes its oxidation reaction by stabilizing its radical cation, resulting in the generation of mobile holes [30–32]. For PTAA, under illumination, a similar mechanism is proposed, whereby the oxidation of PTAA raises the conductivity of the polymer [33]. For both HTLs, the inclusion of tBP promotes a better distribution of the HTL on the perovskite, preventing organic semiconductor/LiTFSI phase segregation [34], thus leading to an improved morphology and uniformity of the resulting layer. However, its unfavorable long-term impact on stability indicates that new doping strategies might be required in the future [35–36]. For this, there have been efforts for finding cheap hydrophobic acidic substances with good solubility in solvents orthogonal to the underlying perovskite active layer. Such an alternative compound is tris(pentafluorophenyl)borane (BCF), which is an electrophilic Lewis acid that interacts with the organic semiconductor and increases its conductivity.

Here, we performed a dedicated study for the HTL/perovskite interface, in order to evaluate the effects of dopants such as BCF on the interfacial potential landscape in working devices. In this work we chose four HTL doping configurations that have been reported for high-performing solar cells [24,37–38]: (i) spiro-OMeTAD doped with LiTFSI and tBP, (ii) spiro-OMeTAD doped with BCF, (iii) PTAA doped with LiTFSI and tBP, and (iv) PTAA doped with BCF. All the cells from all the batches were nominally identical, except for the HTL. We examined the potential distribution in all configurations via KPFM. We cleaved the devices and prepared smooth cross-sections by means of argon ion polishing. To get results that closely simulate the operation of working devices, we used a comprehensive static KPFM measurement protocol (See Supporting Information File 1, Section 2) and measured potential profiles across all layers while applying a voltage or under illumination. Our results indicate that the inclusion of BCF has a passivating effect on iodide defects within the devices. Particularly, a major improvement on the diode character of the HTL/perovskite interface was observed, in both spiro-OMeTAD and PTAA cells. The details of device fabrication, ion milling parameters, and KPFM procedure are reported in the Experimental Section.

## Results and Discussion

### Efficiency characterization

**Figure 1:**
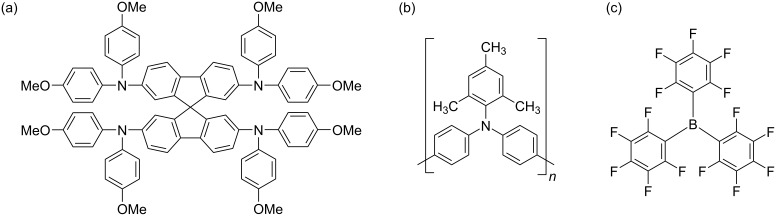
The structures of (a) spiro-OMeTAD, (b) PTAA and (c) BCF.

Whilst BCF (Figure 1) has an advantageous impact on the conductivities of both spiro-OMeTAD and PTAA, when similar dopant concentrations are used, the effect on PTAA is more pronounced, which implies dissimilarities in the underlying doping mechanisms (See Supporting Information File 1, Section 3). Nevertheless, we decided that for our BCF batches the best approach was to dope both spiro-OMeTAD and PTAA solely with BCF and forgoing using further additives LiTFSI and tBP. This was in order to more directly evaluate the effect of BCF compared to the more traditional doping path of LiTFSI and tBP. The BCF concentration used in both cases was 8 wt % with respect to the polymer repeating unit (PTAA), or molecular weight (spiro-OMeTAD).

To confirm the beneficial effect of the doping of the HTL with BCF, we initially characterized the photovoltaic performance of each of the four solar cell batches with a solar simulator under 1 Sun irradiation (1000 W/m^2^). The corresponding parameters are reported in Table 1 and they refer to a statistical analysis of backward scans from ten devices of each batch. A slow scan rate of 60 mV/s was used for the current density–voltage (*J*–*V*) curves so as the ion distribution within the cell is under quasi-equilibrium [39].

**Table 1:** Solar cell device photovoltaic parameters employing different HTL doping strategies.

HTL doping strategy	*J*_sc_ (mA/cm^2^)	*V*_oc_ (V)	FF (%)	PCE (%)

Batch 1: spiro-OMeTAD without BCF	22.3 ± 0.3	0.98 ± 0.15	74.1 ± 0.8	16.2 ± 0.3
Batch 2: spiro-OMeTAD with BCF	23.0 ± 0.8	1.04 ± 0.16	75.6 ± 2.6	17.3 ± 0.4
Batch 3: PTAA without BCF	23.3 ± 0.7	1.00 ± 0.14	75.4 ± 0.9	17.6 ± 0.4
Batch 4: PTAA with BCF	23.6 ± 0.6	1.01 ± 0.12	78.5 ± 0.2	18.7 ± 0.2

We notice that BCF had a beneficial effect on both spiro-OMeTAD and PTAA in terms of photovoltaic parameters. Whilst the positive effect on short-circuit current (*J*_sc_) and open-circuit voltage (*V*_oc_) is marginal, the increase on the fill factor (FF) is more substantial, and is reflected on the elevated average power conversion efficiencies (PCE) of the batches. The average increased efficiency observed in the cells of batches that incorporated BCF can be attributed to the improved conductivity of the HTL material, as well as the passivation of mobile ionic defects. Specifically, these defects are prevented from drifting and accumulating at the interfaces of the perovskite and giving rise to non-radiative recombination sites, which diminish the HTL/perovskite junction quality [40–41]. To investigate the microscopic origins of these effects at the interfaces, we performed cross-sectional KPFM.

**Figure 2:**
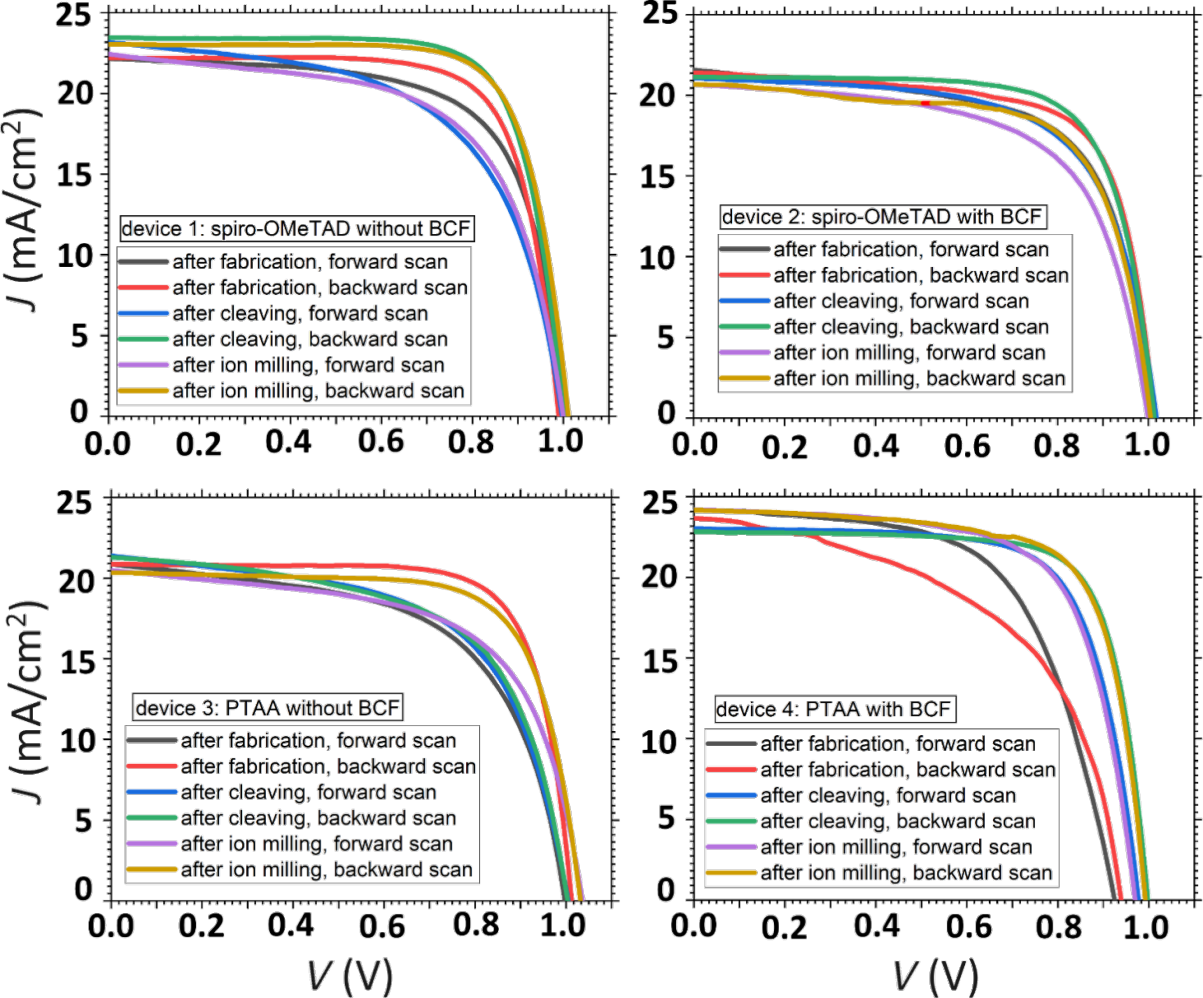
Current–voltage characteristics for the four solar cells that were chosen to be cleaved and polished for cross-sectional KPFM measurements. The plots show that the cells (that were ultimately measured with cross-sectional KPFM), survived both cleaving and consecutive ion milling without significant alteration to their performance. The paradoxically improved performance that is seen in some cleaved or ion milled cells can be attributed to either the well-documented self-healing of PSCs [42], or to difficulties accurately determining the active area of a cleaved solar cell.

A well-performing solar cell was selected from each batch and after cleaving, it was subjected to argon ion milling in order to get a smooth cross-section. This is useful for getting stable KPFM images, without electrostatic cross-talk. At every step of this procedure, the current–voltage characteristics were being monitored, as shown in Figure 2. By carefully selecting the parameters of the ion milling, we can ensure that the exposed interfacial structure is not damaged and the cells remain operational. Additionally, in order to more accurately interpret interfacial measurements, a precise characterization of the positions of the different solar cell layers is required. We identified the thickness and uniformity of the layers by comparing scanning electron microscopy (SEM) and atomic force microscopy (AFM) images (See Supporting Information File 1, Section 4). The lateral resolution for both AFM and SEM measurements is a few nanometers. The AFM channel that exhibited the clearest contrast between the layers was the amplitude error signal during the amplitude modulation topography scan. Supporting Information File 1, Figure S4 and Table S1, show the layered structure and layer thickness for each of the ion polished devices from the four batches. An important note that is highlighted by these measurements is that the optimal HTL thickness indicated by most spiro-OMeTAD PSC recipes is 200–370 nm, whereas that number for PTAA layers is much lower, around 40 nm [24]. The reduced bulk series resistance that comes with a thinner layer is reflected in superior *J*_sc_ values of PTAA cells. On the other hand, thinner HTLs pose a greater challenge in avoiding shunts, which makes device characterization via SEM essential.

To study the effect of different HTLs on the HTL/perovskite interfaces and how their choice affects charge extraction and recombination in our solar cells, we employed cross-sectional KPFM, more specifically our measurement protocol for static KPFM, which allows us to evaluate the response of our cells under both applied voltages and under illumination.

### Kelvin probe force microscopy characterization with an applied voltage

When charges get generated, they drift to the sides of the device to externally recombine, or in the case of open circuit, to accumulate, leading to forward biasing of the solar cell. Therefore, the surface potential profile of a forward biased device can be correlated with the potential distribution under illumination and open circuit [43–45] (See Supporting Information File 1, Section 2). By forward biasing, we bypass the open-circuit conditions and have a continuous charge flow within our devices, which operate with an external source of voltage. Consequently, charge transport can be studied, which depends on the diode characteristics of the interfaces. By biasing our devices with a voltage value close to *V*_oc_, we can plot the potential distribution across the layers of our solar cells and evaluate the charge extraction at their interfaces.

**Figure 3:**
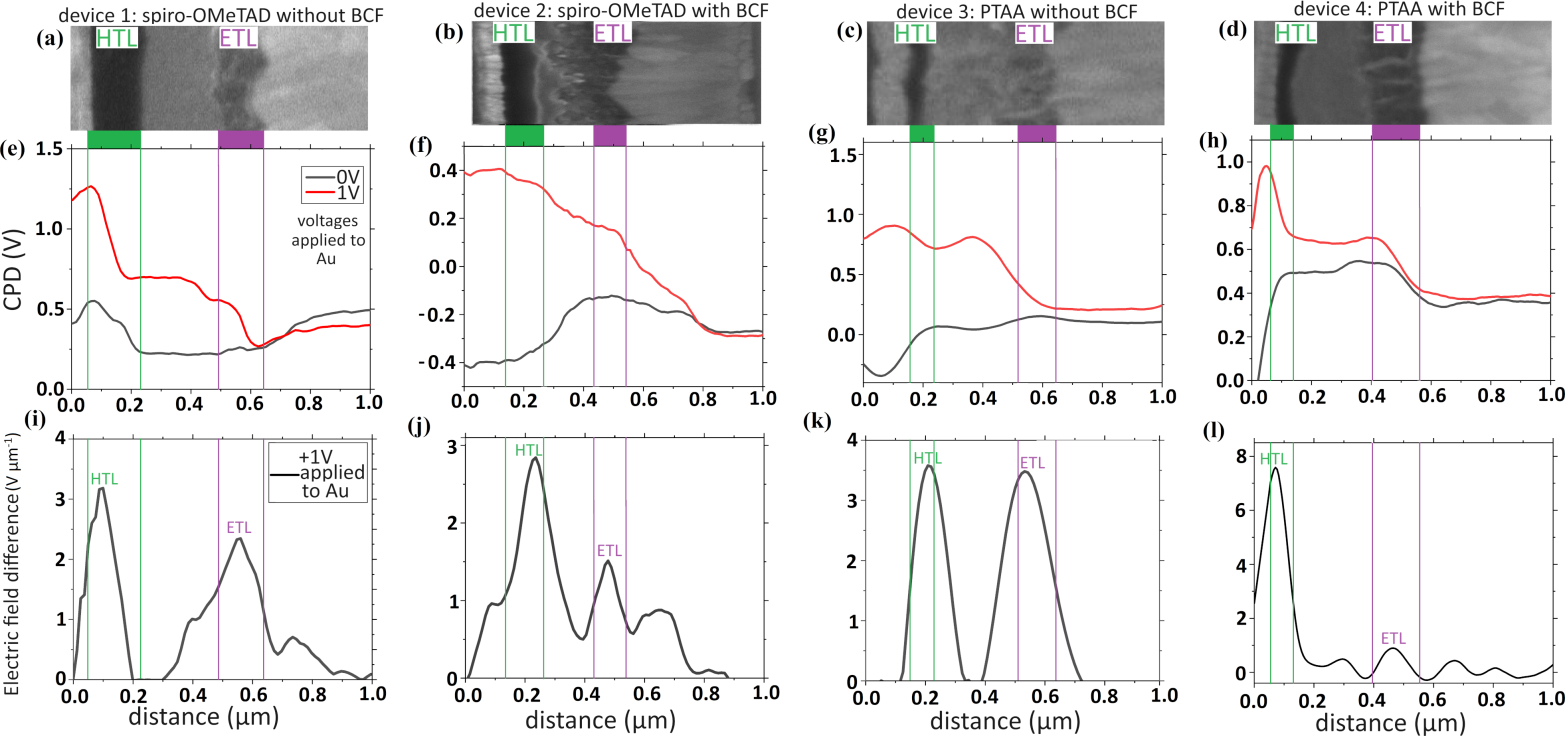
(a–d) SEM images showing the position of the hole and electron transport layers (HTL, ETL). (e–h) The CPD results of the cross-sectional KPFM measurement under dark/short circuit (black curves) and with an applied voltage of +1 V on the Au side (red curves, FTO side grounded). We apply +1 V because *V*_oc_ ≈ 1 V. (i–l) The electric field difference built-up at the perovskite interfaces induced by the applied voltage.

The CPD profile graphs under dark and short circuit depend on the relative work function of the materials comprising the different layers of the devices. In particular, the CPD value of the perovskite layer is influenced by the composition and the doping of the perovskite [15,46]. In Figure 3, the potential profiles plotted for the four devices exhibit features that deviate from the ideal profiles of a p-i-n junction (See Supporting Information File 1, Section 1), with voltage drops and rises being apparent because of the band bending introduced by mobile ions or surface defect states caused by the cleaving. Furthermore, the CPD decrease (black curves) on the HTL side relative to the perovskite when BCF is included in both cases reveals the p-doping of the HTL by the Lewis acid [47], whilst the increase of the perovskite CPD indicates an indirect n-doping induced by BCF. When subjecting the devices to a forward bias of 1 V (red curves), which is approximately the value of the open-circuit voltage, we were able to observe potential profile distributions indicative of p-i-n junctions in all devices, with a lower CPD on the ETL side where the electrons accumulate under bias. Subtracting the first measurement from the second, we filter out all information from the data that does not pertain to potential changes due to charge separation and accumulation at the perovskite junctions because of the applied bias (like the aforementioned defect-state and relative work function contributions).

Consequently, we use these bias-induced potential profiles (that result from the aforementioned subtraction of CPD profiles) to plot electric field profiles (Figure 3i–l) that reflect the junction quality of the perovskite absorber with its adjacent transport layers. We chose to present the results as electric field differences and not as bias-induced voltage profiles, since the electric field peaks provide a more intuitive way of immediately identifying the position of the built-in fields that enable charge separation. These profiles that are being referred to as “electric field difference” are presented in Figure 3i–l.

To extract the electric field difference profiles, we applied the following equation:


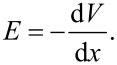


Here, *E* is the electric field difference, *V* is the measured potential under 1 V bias minus the potential at dark/short-circuit conditions and *x* is the distance.

**Figure 4:**
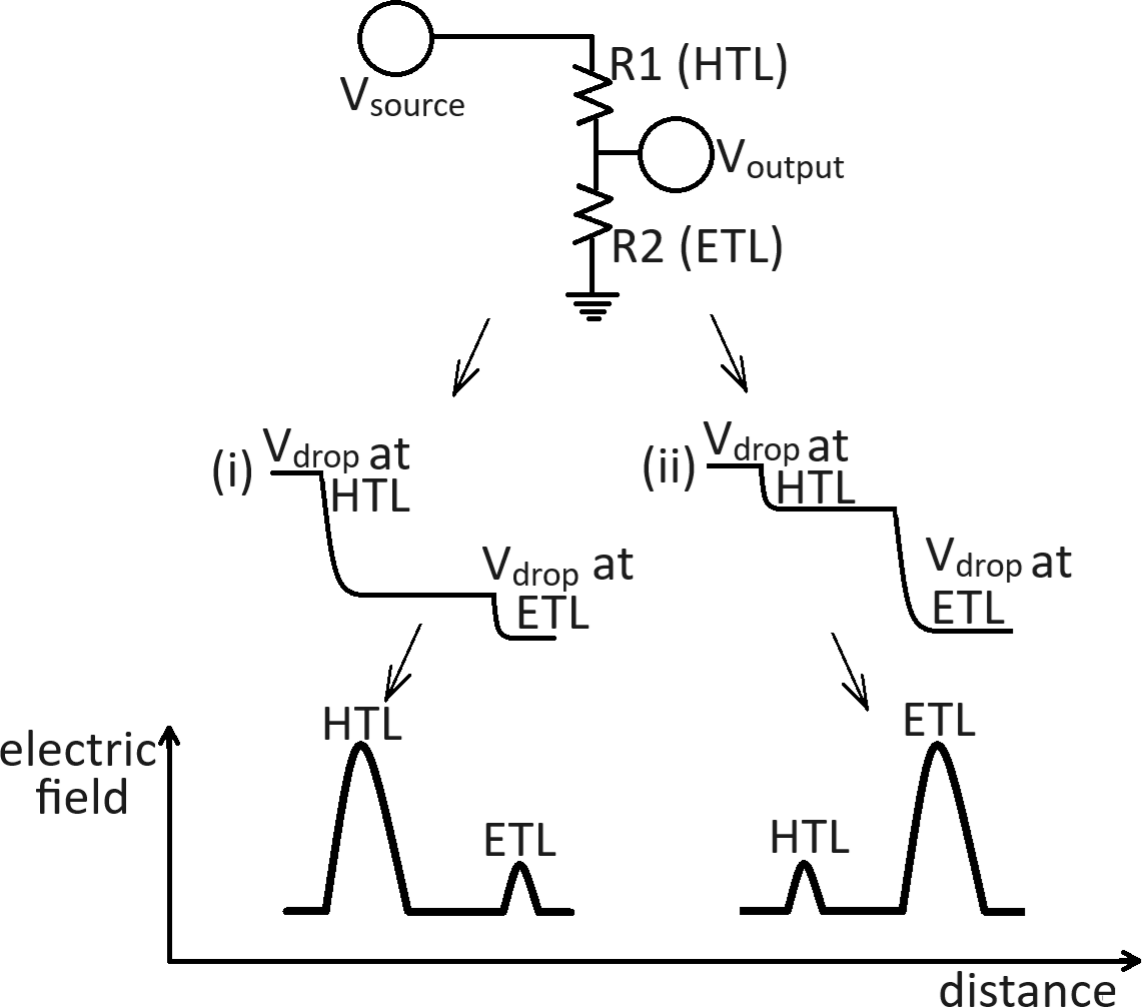
A schematic depicting a simple representation of the two interfacial resistances of the perovskite diode-junctions, as resistors in a voltage divider. Whilst KPFM is a more complex experiment, we can juxtapose the two measurements because they both result in quantifying the potential characteristics of a system, where current flows through resistive elements. A resistor represents the ability of a diode junction to efficiently block current in reverse bias. (i) If R1 > R2 (here: HTL shows better rectifying properties and a better diode quality than ETL), the voltage mostly drops at R1 (HTL). (ii) If R1 < R2 (here: HTL exhibits a more ohmic behavior than ETL due to a decreased charge extraction and more interfacial recombination), the voltage mostly drops at R2 (ETL). Given the identical ETLs of the four device batches we fabricated, we can directly compare the four different HTLs we used. The potential profiles are measured with cross-sectional KPFM and the electric field profiles are derived by the equation *E* = −d*V*/d*x*, since electrostatic fields are conservative.

For these measurements, we applied +1 V to the Au electrode to forward bias the device (Figure 3e–h and Supporting Information File 1, Section 5). The resulting magnitude of the electric field difference profile reflects the relative competition of the two junctions on either side of the perovskite to efficiently extract charges [48], as described by our model in Figure 4.

By applying a forward bias of approximately *V*_oc_, we bring our cell into the same configuration as the open-circuit and illuminated case and we have a sufficient diffusive current flowing through the two junctions, but smaller in magnitude compared to the current flowing through an ohmic contact. Therefore, the junction exhibiting the more pronounced rectifying behavior will still limit the current flow. For our model, the influence of the resistance of the active layer is omitted, as it remains the same for all devices tested. If the rectifying capability of the HTL/perovskite junction is poor, then under the applied bias, more current will readily flow through it and the voltage will mainly drop on the ETL interface, where the diode quality is better and less saturation current flows. This larger voltage drop corresponds to a larger electric field difference magnitude on the ETL side (Figure 4).

Any improvement in charge extraction at one interface does not necessarily increase the total (bias-induced or photo-) voltage, but rather, it redistributes how this voltage is shared between the HTL and ETL interfaces (for the following explanation bias-induced voltage and photovoltage can be thought of as equivalent, as explained in Supporting File 1, Section 2). That is because under open-circuit conditions, the total photovoltage across the device is fundamentally limited by the quasi-Fermi level splitting (QFLS) in the perovskite absorber, meaning that the overall voltage is mainly constrained by the recombination processes in the absorber. An improved interfacial behavior cannot overcome bulk recombination limits, even though poor alignment at defective interfaces creates additional losses that compound the problem. Our results show that under equilibrium, an improved HTL (better energy alignment with the perovskite, reduced interfacial recombination) only has a secondary effect on QFLS, which means that bulk recombination predominantly defines the V_oc_.

Since our data (Table 1) indicates that *V*_oc_ remains mainly unchanged after replacing the original HTLs with BCF including ones, this suggests that the total QFLS does not increase with the inclusion of BCF, in both the spiro-OMeTAD and PTAA cases. However, the improved FF in both types of cells (Table 1) reveals that BCF doping plays a critical role in improving the charge transport and interfacial properties of the devices. Firstly, by increasing the conductivity of the organic compound it is doping, BCF reduces the series resistance of the HTL and improves charge transport to the terminal. Furthermore, as a strong Lewis acid, BCF passivates mobile iodide defects at the perovskite/HTL interface, which act as recombination centers, thus reducing non-radiative recombination losses and improving hole extraction efficiency. These beneficial effects lead to a redistribution of the voltage drops at the HTL and ETL junctions, with them being increased and decreased respectively, while the total voltage drop is maintained around the value of *V*_oc_. All the above become apparent when plotting the electric field difference profiles (Figure 3i–l), by differentiating the bias-induced voltage profiles, where the aforementioned voltage drops are now expressed as electric field peaks.

For the interpretation of the electric field peaks, both their widths and their heights should be taken into consideration, as they both define the area under the peaks, which corresponds to the total voltage drop across the interfaces of the perovskite layer. Specifically, the peak widths depend on the span of the voltage drop, which depends on the thickness of the transport layers, which is variable along the cell, (as can be seen in Figure S4 and Supporting Information File 1, Section 6). Since our graphs refer to just a specific line across the cell layers, that might be a source of inconsistency for the plotted peak width. Correspondingly, that affects the peak height, since a broader voltage drop would give a smaller height of electric field for the same drop magnitude. Therefore, for a complete understanding of the electric field difference plots (Figure 3), an analysis of their integrals which take into account both the peak heights and widths is required (see Supporting Information File 1, Section 7). Results show that the areas under the electric field peaks of the HTL side are smaller than those on the ETL side for devices 1 and 3, whereas the peaks of the HTL side shows a larger area than that of the ETL side for devices 2 and 4. This shows that, given that the ETLs remained the same, the BCF dopant had a beneficial effect on the rectifying properties of the HTL/perovskite junction, in accordance with our proposed explanation regarding the improvement of the HTL properties when BCF is added.

Another problem may arise from the fact that the perovskite layers do not adhere completely smoothly and uniformly to the transport layers, so whilst they have statistically similar widths in the four devices, for a specific measurement, the junction distances may vary between the four solar cells. Also, whilst the electric field peaks are indicative of the diode junction positions, these may not coincide with the two perovskite interfaces, since the exact position where charge extraction takes place might be affected by factors such as ion accumulation. In a previous study, we demonstrated that the position of the potential drop can be significantly shifted into the transport layers, due to ionic interactions [15].

In the case of the cells incorporating spiro-OMeTAD, from the *J*–*V* characterization, we expect the potential profiles of the cells with BCF to reflect the increased efficiency compared to the ones with LiTFSI/tBP. Indeed, from Figure 3i,j we can see that in the spiro-OMeTAD cell without BCF, the ETL/perovskite junction exhibits a marginally better diode quality relative to that of the HTL/perovskite interface. This is due to the fact that even though its peak is comparatively smaller in magnitude, the area under that peak is slightly larger, as shown in Supporting Information File 1, Figure S7. However, the situation reverses in the cell from the BCF batch. A diode with higher quality at the HTL side leads to a more efficient charge extraction/charge blocking on that interface relative to the other. Therefore, there is a larger and broader electric field difference peak due to the higher value of extracted charges at that interface. A similar circumstance arises in the PTAA solar cell when LiTFSI/tBP is replaced by BCF. Then, we notice a strong increase in the HTL field strength relative to the ETL, which reflects the improvement in the HTL/perovskite diode quality when BCF is incorporated (Figure 3k,l and Supporting Information File 1, Figure S7). This result is associated with improved charge transport properties and a reduction in the number of trap states at that interface. We propose that BCF, as a Lewis acid electron acceptor, efficiently coordinates with under-coordinated iodide defects and passivates them, increasing junction quality, promoting p-doping, and diminishing recombination at the HTL interface. This, in conjunction with the superior PTTA/perovskite interaction and the favorable morphological properties of PTTA, leads to a considerable increase in the voltage drop at the hole extracting side of the device.

We also noted the iodide passivation as a main source for the discrepancy between the CPD profiles of devices 1–2 and 3–4 in Figure 3e–h, as the existence of iodide ions and their accumulation at the interfaces has an effect on the measured potential profiles.

### Kelvin probe force microscopy characterization under illumination and open circuit

To study the quality of the HTL interfaces regarding recombination of photo-generated charge carriers, we illuminated the solar cell under open-circuit conditions and subtracted the dark/short-circuit profile, in order to extract the photo-carrier voltage. The resulting profile is independent from effects coupled to the built-in field, as well as from the aforementioned contributions of the relative work functions of the materials and possible surface defect states created from cleaving (See Supporting Information File 1, Sections 8 and 10). This time, the voltage is generated within the active area of the solar cell and the charge carriers are induced by the illumination. Unlike the previous experiment, where we considered charge transport as the reason for our results, we now force our devices to operate in open circuit under a net zero charge flow condition. Therefore, charge recombination becomes the limiting factor that defines *V*_oc_ and device performance.

For devices 1 and 3 (without BCF), we can identify two diode junctions on either side of the perovskite absorber, which are almost equal in magnitude, whereas in devices 2 and 4 (with BCF) the HTL/perovskite junction clearly becomes the dominant one, as shown in Supporting Information File 1, Figure S10. Judging from the photo-charge built-up at the interfaces of the perovskite layer, for the solar cells that incorporate LiTFSI and tBP, there is not a single operation defining voltage drop, but rather both perovskite interfaces are approximately equal in their voltage drop magnitude and therefore contribute equally to charge extraction. Devices that exhibit two charge separating junctions are more prone to charge recombination compared to devices with just one junction [46]. On the contrary, devices that incorporate BCF-doped HTLs, exhibit one large drop at the perovskite/HTL interface, indicative of the dominant diode junction that exists there. In order to further understand charge separation within the solar cells, we can use these photopotential profiles in order to examine charge extraction and accumulation within the solar cells.

By plotting charge density profiles we can more clearly point out the sum of photo-charge that has been extracted at the perovskite interfaces and accumulated under open-circuit conditions. Unlike the measurements under bias, here we excited a large number of charges within the absorber, which diffuse, get extracted, and aggregate at the interfaces, giving rise to a large charge density magnitude we can plot. In order to generate the photo-carrier density profiles, we applied the Poisson’s equation:


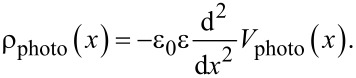


Here, ε_0_ is the permittivity of free space, ε is the relative permittivity of the perovskite material, and *V*_photo_ is the photopotential profile measured with KPFM.

Under open-circuit conditions, photo-generated free carriers are generated within the absorber material and diffuse to their corresponding side of the cell: electrons towards the ETL interface and holes towards the HTL interface. The relative ability of these interfaces to efficiently extract (and block) charges depends on the energetic alignment with the perovskite and the defect-induced interfacial recombination that occurs there. These factors determine the charge density that will ultimately accumulate on the cell edges under open-circuit conditions.

In Supporting Information File 1, Figure S11a–d we can identify that in the case of the spiro-OMeTAD cell, there is an increase in the perovskite dark CPD, indicative of n-type doping, indirectly induced by the BCF additive. For the PTAA cell, the perovskite dark CPD also exhibited an increase relative to the CPD of the HTL. When the illumination is turned on, the BCF-doped spiro-OMeTAD cell exhibits a linear CPD, indicative of a homogeneous electric field within the perovskite and a p-i-n junction, where charges can drift inside the perovskite to the corresponding interfaces. We have reported such uniform potentials, without significant local variations, in a previous study [15]. In the case of the PTAA cell, the CPD within the perovskite remained flat, indicating that the charge carriers have to diffuse to the interfaces and separate under the influence of the local fields there. In addition, when BCF was introduced in both spiro-OMeTAD and PTAA cells, the open-circuit photovoltage built up more strongly at the HTL/perovskite interface. This indicates the increased charge separation potency of the junction due to decreased charge recombination rates (Supporting Information File 1, Figure S10).

**Figure 5:**
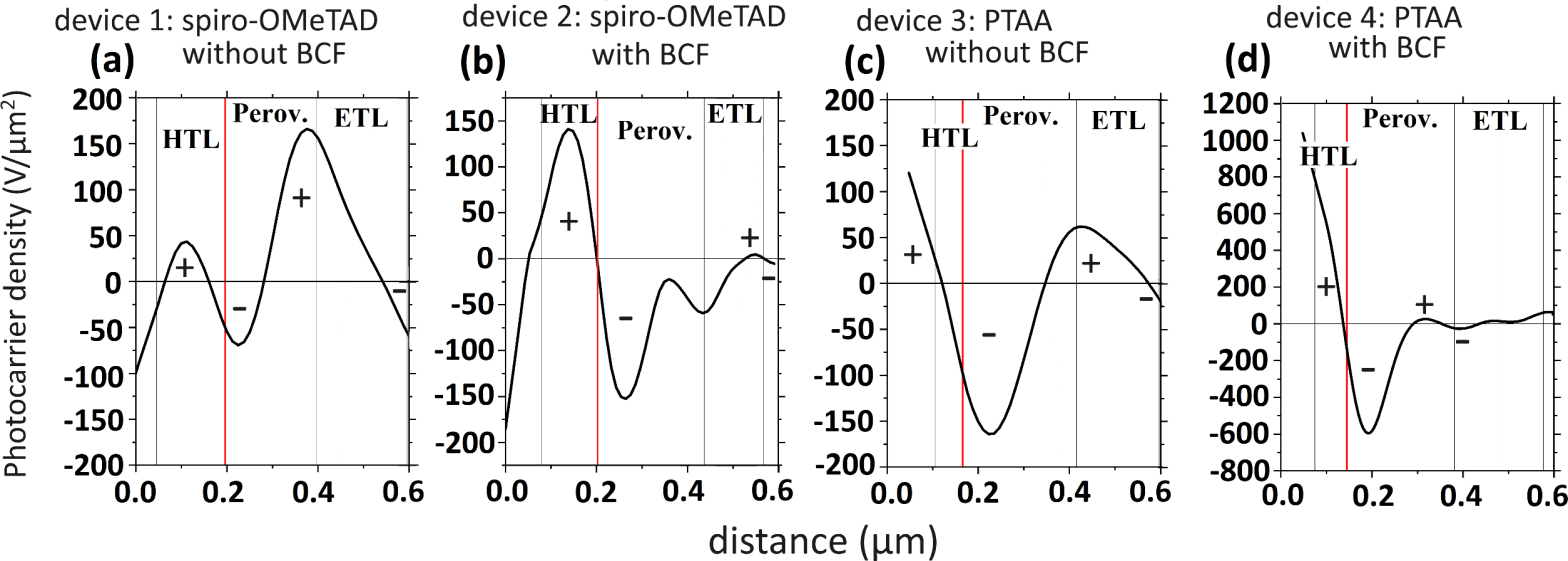
(a–d) Plots of the photo-charge density profiles (ρ_photo_(x)/ε_0_ε) across the three inner device layers (HTL, perovskite, and ETL), with notations for positive and negative charge accumulation under open-circuit conditions. The full graphs are presented in Supporting Information File 1, Figure S11.

In Figure 5a–d we again identified the charge separating junctions in each cell, as calibrated by the AFM and SEM images (Supporting Information File 1, Figure S4). In the cells that use spiro-OMeTAD as HTL, we can identify that positive charges get separated at the HTL/perovskite interface, whereas negative charges get separated within the mesoporous TiO_2_. In the traditionally doped cell, we notice that on the HTL side there is a comparable amount of electrons and holes on each side of the junction. On the ETL side, however, there are more positive charges on the perovskite side than ETL-extracted negative charges, which leads to a positive charging of the perovskite. On the contrary, when BCF is added in the spiro-OMeTAD precursor solution, the HTL/perovskite interface extracts charges more efficiently and becomes the dominant junction relative to the one on the ETL side. A previous study of our group [15] has associated the charging within the perovskite absorber under open-circuit conditions with unbalanced recombination rates of electrons and holes at its two interfaces. More specifically, a positive charging of the perovskite was connected with a preferential recombination of electrons at the HTL side. The elimination of this magnitude of positive built-up indicates that BCF has improved the junction quality in terms of charge carrier leakage. In addition, it has diminished defect-induced interfacial recombination by passivating iodide interstitials within the perovskite, which can transport to the interfaces and act as non-radiative recombination centers [49–50].

In the cells that use PTAA/BCF as HTL, we noticed a large increase in the number of charges that get separated at the HTL junction compared to the ETL junction (in relation with the traditionally doped PTAA cell). This indicates an improvement in the junction quality on the HTL side enabled by the BCF. Again, we propose that BCF is forming a Lewis adduct with under-coordinated halide ions that have migrated towards the HTL side and passivates them, diminishing interfacial recombination and increasing charge extraction [51–52]. In both cases of spiro-OMeTAD and PTAA cells, the charge magnitude at the HTL interface overtook that at the ETL interface, which is proof of the increased HTL/perovskite junction quality and reduced recombination rates when BCF is included as additive.

Moreover, in the past, our group proposed the existence of an interlayer between HTL and perovskite, created by spiro-OMeTAD – iodide complex formation, which reduced the efficiency of solar cells [15]. Results showed that this was visible in cross-sectional KPFM results by way of a slight shift (≈70 nm) of the interfacial electron blocking layer into the spiro-OMeTAD. This interaction was said to de-dope spiro-OMeTAD and introduce a resistive layer that acted as a barrier for charge extraction. Such interaction between spiro-OMeTAD and iodide ions, as well as PTAA and iodide ions has also been reported elsewhere in the literature [53–54]. In Figure 5a–d (vertical red lines) we can see that both devices that do not include BCF exhibit this shift of the electron blocking interface (≈40 nm for the spiro-OMeTAD device, ≈45 nm for the PTAA device), which indicates the negative interaction of the mobile iodide defects that have diffused towards the hole extracting interface. On the contrary, the devices that incorporated BCF do not exhibit such shift, which suggests the successful passivation of iodide defects by the Lewis acid. We note that the spatial resolution of cross-sectional KPFM is sensitive enough to distinguish these slight shifts of tens of nanometers. This microscopically observed result translates to the macroscopic efficiency characterization, specifically the increased FF, which directly relates to a decrease in series resistance close to the HTL side of the corresponding devices.

## Conclusion

In conclusion, we incorporated BCF, an electrophilic substance with passivating properties, in the two most popular HTL semiconductors for PSCs. Current–voltage characterization indicated that the inclusion of BCF had a beneficial effect on the performance of both spiro-OMeTAD and PTAA cells. By applying our comprehensive static cross-sectional KPFM measurement protocol, we showed an increased junction quality and a reduced recombination rate for the HTL/perovskite interface of the selected characteristic devices from the batches that included BCF, compared to the ones from the batches that used the traditional doping method. Furthermore, for the devices that incorporated BCF, there is strong indication that the Lewis acid has a passivating effect on iodide defects, which accentuates the positive impact of BCF as an HTL additive for PSC performance enhancement. Cross-sectional KPFM provides a valuable tool for locally evaluating that impact and our set of measurements can act as a standard for evaluating devices for individual layer optimization.

## Experimental

### Solution and device preparation

For device fabrication, we mainly used the recipe of Klasen et al. [21]. We patterned fluorine-doped tin oxide (FTO) substrates on thin (1.1 mm) glass from Ossila (11–13 Ω/cm^2^) with Zn powder and a 2 M HCl solution. Then, we brushed it thoroughly using a liquid alkaline concentrate (Hellmanex), followed by a 30 min argon plasma cleaning (200-G TePla Plasma System, Technics Plasma GmbH, at 0.14 mbar and 280 W). Consequently, we deposited a compact layer of TiO_2_ via an aqueous 0.75 M TiCl_4_ solution (Sigma-Aldrich, 99.99% trace metal basis). A volume of 80 μL of the solution was spin-coated at 5000 rpm for 30 s, and the resulting films were annealed at 500 °C for 30 min. Afterwards, we deposited a mesoporous TiO_2_ layer from a (transparent) titania paste solution (Aldrich, 16.67 wt % in ethanol) via spin coating and annealed it (same parameters as in the previous step). After each of these titania deposition steps, we subjected the films to a UV-ozone cleaning step (FHR UVO 150) for 30 min, with an oxygen flow of 10 L/min. Then, a 1 M methylammonium lead iodide (MAPI) precursor solution was prepared (lead iodide 99.99% trace metals basis from TCI, methylammonium iodide >99.99% from Greatcell solar) with the materials dissolved in a DMF/DMSO (4:1) solvent and spin-coated using a two-step deposition method (500 rpm for 10 s and 4000 rpm for 25 s). A volume of 150 μL of toluene was used as anti-solvent 10 s into the second step. The perovskite was crystalized during a 100 °C annealing step for 30 min. For the cells that incorporated spiro-OMeTAD, we used a solution containing 72.3 mg spiro-OMeTAD, 28.8 μL tBP, and 17.5 μL LiTFSI solution (520 mg in 1 mL acetonitrile), all dissolved in 1 mL chlorobenzene (or BCF in chlorobenzene at an 8% mol ratio with spiro-OMeTAD, for the corresponding devices) and spin coated 80 μL at 4000 rpm for 30 s. For the cells that incorporated PTAA, we used a solution containing 15 mg PTAA, 7.5 μL, LiTFSI solution (170 mg in 1 mL acetonitrile), and 7.5 μL tBP solution (1:1 in acetonitrile), dissolved in 1 mL toluene. For the BCF batch, instead of LiTFSI and tBP, BCF was added in at an 8% mol ratio to PTAA. After the HTL deposition, an Au electrode was evaporated as a back contact under vacuum (Edwards FL 400 Au evaporator). The devices were characterized in terms of efficiency with a solar simulator (Abet Technologies, SunLite) under AM1.5 illumination.

#### Cross-section preparation

To create solar cells with exposed cross-sections, we mechanically cleaved the solar cells along the direction perpendicular to their active layers, thus exposing their interfaces for direct measurement. In order to get a smooth cross-section, we employed argon ion milling (Hitachi IM4000, discharge current: 130 μA, acceleration voltage: 2.5 kV, discharge voltage: 0.75 kV). Since argon is inert and the process occurs under vacuum, we minimized the possibility for chemical contamination of our solar cells.

#### Kelvin probe force microscopy

Mapping the surface potential of the samples was conducted via an Asylum Research MFP3D microscope (Oxford Instruments) and an HF2LI-MOD lock-in amplifier (Zurich Instruments), in an argon atmosphere glove box (less than 1% ppm O_2_ and negligible humidity). The cantilever used was SCM PIT V2 (resonance frequency: 75 kHz, spring constant: 3 N/m, Bruker). The scan rate of the measurement was 0.5 Hz. To increase the reliability of our data, we employed heterodyne-KPFM [55], whereby we mechanically excited the cantilever at its first resonant frequency, *f*_1_, and electrically excited at a frequency of (*f*_2_–*f*_1_), where *f*_2_ is the second resonant frequency [56]. Frequency mixing between the mechanical vibration at *f*_1_ and the electrostatic force generates a sideband signal at frequency *f*_2_, which is used as input for the KPFM feedback loop. For the extraction of the electric field and photocarrier density profiles from the surface potential data, we applied the definitional voltage equation for a conservative electric field and the Poisson equation, respectively. The profiles were smoothed with a 30 point adjacent-averaging method, to get smooth derivative curves with negligible noise.

## Supporting Information

File 1 Additional information.

## Data Availability

Data generated and analyzed during this study is available from the corresponding author upon reasonable request.
