# Supplementary material for: The impact of tris(pentafluorophenyl)borane hole transport layer doping on interfacial charge extraction and recombination
Source: Beilstein J Nanotechnol. 2025 May 21;16:678–89. doi: 10.3762/bjnano.16.52 (PMC12117211; doi:10.3762/bjnano.16.52)
Supplement: File 1 — Additional information. [file Beilstein_J_Nanotechnol-16-678-s001.pdf]

BEILSTEIN JOURNAL OF NANOTECHNOLOGY

Supporting Information

for

The impact of tris(pentafluorophenyl)borane hole transport layer doping on interfacial charge extraction and recombination

Konstantinos Bidinakis and Stefan A. L. Weber

Beilstein J. Nanotechnol. **2025,** *16,* 678–689. doi:10.3762/bjnano.16.52

Additional information

License and Terms: This is a supporting information file under the terms of the Creative Commons Attribution License (https://creativecommons.org/licenses/by/4.0). Please note that the reuse, redistribution and reproduction in particular requires that the author(s) and source are credited and that individual graphics may be subject to special legal provisions.
The license is subject to the *Beilstein Journal of Nanotechnology* terms and conditions: (https://www.beilstein-journals.org/bjnano/terms)

Section 1: Energy band diagram of a perovskite solar cell (PSC)

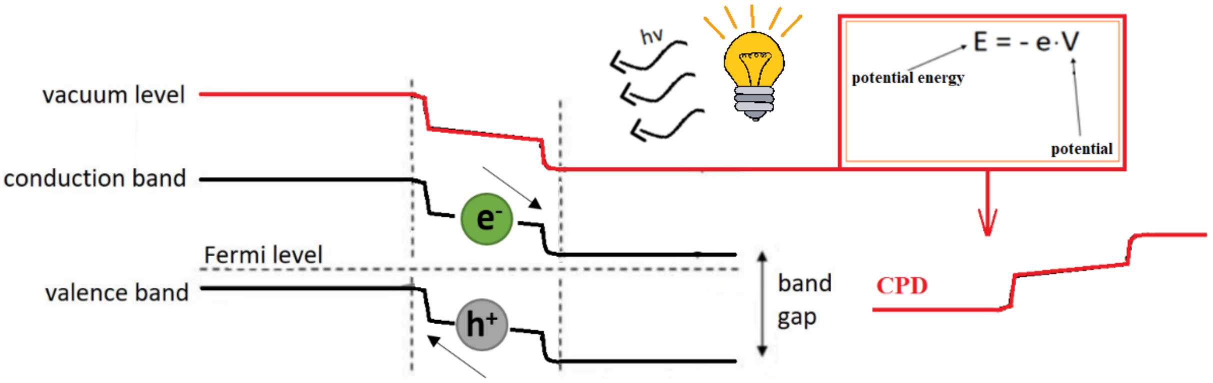

Figure S1: A simplified energy diagram of a p-i-n junction of a PSC with its layers under electrical contact just before we turn the illumination on. A contact potential difference (CPD) profile line measured with Kelvin probe force microscopy (KPFM) expresses the mirror image of the shifted vacuum level with respect to the horizontal axis. With this measurement, we can identify the built-in fields at the junctions of the perovskite. In principle, the resulting profile reflects the relative work functions of the different materials of the device layers, which give rise to built-in fields at the interfaces. After the illumination is on, the photo-generated charges move under the influence of these built-in fields.

Section 2: Measurement protocol for devices under bias and under illumination

In order to evaluate the performance of our solar cells we demonstrate a simple representation of the solar cell as a p-i-n junction, with a depletion and an accumulation layer at each side of the perovskite active layer (Figure S2). The first KPFM measurement involves the illumination of the sample with a light source that resembles 1 Sun illumination. The photogenerated charge carriers drift along the perovskite and are extracted at the corresponding interface (holes at HTL and electrons at ETL). Under open-circuit conditions, these charges cannot externally recombine and therefore accumulate at the two junctions of the device. This measurement allows us to see where the extracted photo-charges end up within the device and draw conclusions about the potency of charge extraction at the junctions and whether the performance is hindered by interfacial charge recombination. This newly established charge distribution affects the surface potential that we measure with KPFM. In addition, we conduct the same measurement but with the application of an applied voltage instead of illumination. In this instance, we do not excite photocharges within our absorber, but rather simulate the resulting charge configuration of our system by applying a voltage with a value close to V_{oc} (Figure S3). This measurement allows us to judge the diode quality of the junctions between the perovskite and its two adjacent charge selective layers. For both these measurements, we take potential profile lines across the whole device under dark and short-circuit conditions. These measurements are used as a baseline that we subtract from any consecutive “under illumination” or “with applied voltage” measurements. With this action, we ultimately decouple our results from the relative work functions of the materials comprising the layers and from the effect of surface states or other imperfections that affect every KPFM measurement, regardless of applied conditions. Therefore, the resulting potential profile lines are the result of only photogenerated charges (“under illumination” measurement), or charges

that have drifted, been extracted, and accumulated because of the applied field ("with applied voltage" measurement).

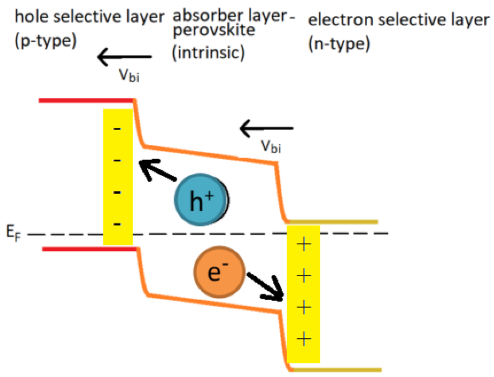

Figure S2: An idealized depiction of the p-i-n junction of a perovskite solar cell. We assume that the equilibration of the Fermi levels of the perovskite with the charge selective layers leads to the formation of an accumulation region between the perovskite and the HTL and a depletion region between the perovskite and the ETL. The corresponding electric fields drive charge extraction at these two junctions.

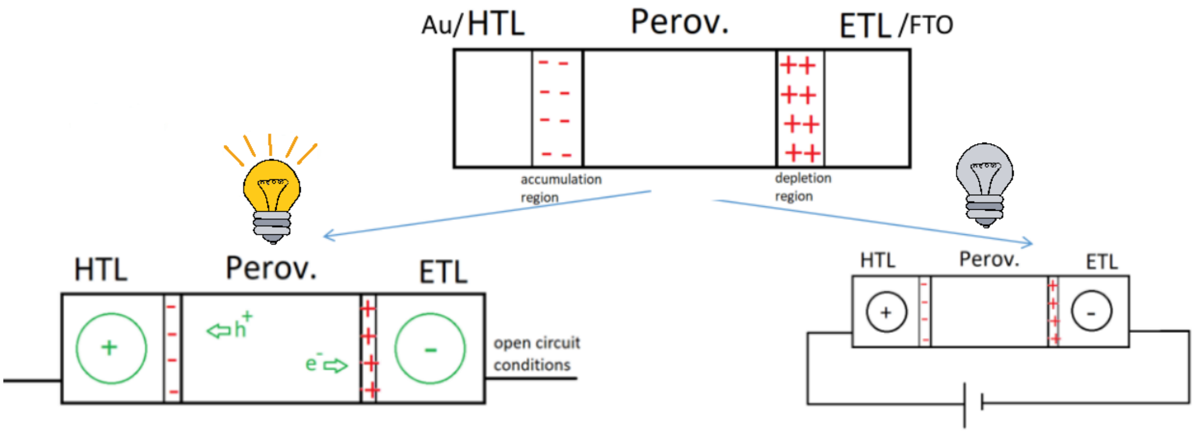

Figure S3: A schematic depiction of the two cross-sectional measurements we carried out. With the "illumination/open-circuit" measurement, photogenerated charges accumulate and screen the two built-in fields at the perovskite junctions. Since no charge transport takes place under open-circuit, the limiting factor for this process is charge recombination. With the "applied voltage" measurement, we simulate the aforementioned charge accumulation by forcing it on our solar cell via an external

applied voltage of approximately V_{oc}, since under open-circuit, electrons accumulate on the FTO side of the device and holes on the Au side. In order to mimic that effect in absence of illumination and simulate the resulting forward biasing of the cell, we need to apply a positive voltage to Au, or a negative voltage to FTO. The applied voltage needs to be ≈ V_{oc}. With this measurement, we do not generate photocarriers, but drive the existing intrinsic ones towards the interfaces. In this measurement, charge transport takes place, so we can evaluate the effect of junction quality at the interfaces on charge extraction. In Figure S3, the circles with the positive and negative signs represent the accumulated positive and negative charges.

Section 3: Doping mechanisms of HTL using BCF and fabrication details

For the interaction of the BCF dopant with the organic semiconductors, two distinct paths have been proposed. The first one predicts the formation of a Lewis acid/Lewis base complex, by a dative covalent bond between the nitrogen of the organic semiconductor and the boron of BCF. The other mechanism is described by the ground-state integer charge-transfer model, whereby we get free ions, increasing the conductivity. Several attempts to incorporate BCF into spiro-OMeTAD and PTAA are reported, indicating a beneficial effect to the resulting performance and stability of the cells. The proposed doping mechanism when PTAA is the material of choice involves the partial charge transfer between PTAA and BCF, which due to steric hindrance, forms a frustrated Lewis acid/Lewis base adduct between the two compounds [1-3]. Recently reported research suggests that the addition of BCF to PTAA leads to better charge carrier mobility for the HTL, as well as better performance and stability for the corresponding devices, compared to the traditional Li salt doping method [4]. The optimal dopant molar fraction for BCF in PTAA is around 10 wt %, with groups reporting their best performance with a 5 wt % [4], 8 wt % [5], and 12 wt % [6] BCF solution. Efforts that incorporate BCF in PTAA predominantly forgo the Li salt/tBP additives, which stems from the high surface quality and low rates of interfacial recombination at the PTAA/perovskite interface [7]. This shows that as long as PTTA is effectively doped by an additive, the physical properties and charge-extracting capability of its HTL junction are going to suffice for a high performing cell. Doping is also needed when spiro-OMeTAD is used as HTL, because despite spiro-OMeTAD films showing a reasonable hole mobility, they lack in overall conductivity. Therefore, an increase in their charge carrier density is needed. Li et al. reported that even though the addition of acids as dopants in spiro-OMeTAD increases the overall conductivity of HTLs and performance of devices, the acids alone cannot efficiently oxidize spiro-OMeTAD;

therefore, the inclusion of the LiTFSI and tBP co-dopants is still required [8]. The reason for this is that while BCF can oxidize spiro-OMeTAD, it cannot stabilize the spiro-OMeTAD cation [9,10]. In addition, it is known that LiTFSI p-dopes the organic semiconductor by keeping its radical cations, which have resulted from oxidation, in a stable state [11]. Additionally, a different doping mechanism of spiro-OMeTAD has been proposed when BCF is used as a dopant, which, unlike in the PTAA/BCF case, involves the complete charge transfer between molecule and dopant, and the complete ionization of spiro-OMeTAD [9]. For spiro-OMeTAD, it is debated whether BCF necessarily needs to be used in conjunction with Li salt and tBP, with some results showing that the conductivity of the resulting HTL increases more optimally when Li salt and tBP are also included [9], whilst others showing that BCF can work well on its own as an outright LiTFSI/tBP substitute [12]. The optimal dopant molar fraction of BCF in spiro-OMeTAD reported by Ye et al., similarly to the PTAA case, was between 5–10 wt % when Li salt/tBP co-dopants were also used [9], whereas Liu et al. reported a value closer to 12 wt % when they were not [12].

Section 4: Calibration of layer thickness with SEM and AFM

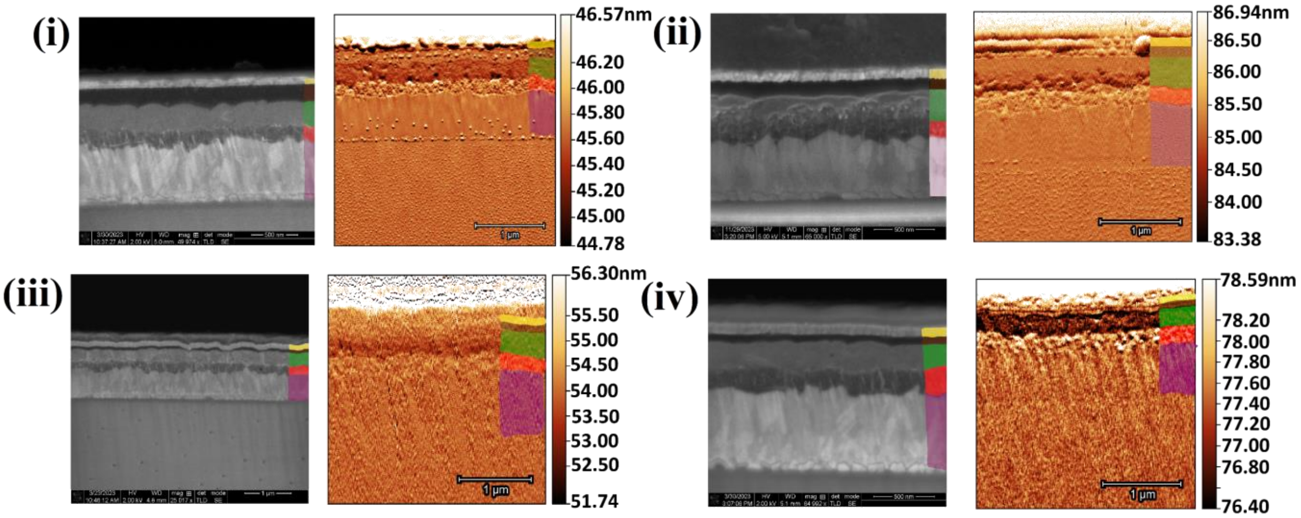

Figure S4: SEM and AFM (dynamic mode, amplitude channel) images for device with: (i) spiro-OMeTAD without BCF, (ii) spiro-OMeTAD with BCF, (iii) PTAA without BCF, and (iv) PTAA with BCF.

Table S1: Thicknesses of the layers as evaluated from SEM and AFM images, for each measured device.

Layer thickness (nm)	1: spiro-OMeTAD without BCF	2: spiro-OMeTAD with BCF	3: PTAA without BCF	4: PTAA with BCF
Au electrode	100	79	106	75
spiro-OMeTAD	150	124	60	70
perovskite	230	234	250	240
mp/c-TiO_2	170	130	181	200
FTO electrode	620	510	570	610

Section 5: KPFM results of forward biased devices from both Au and FTO sides

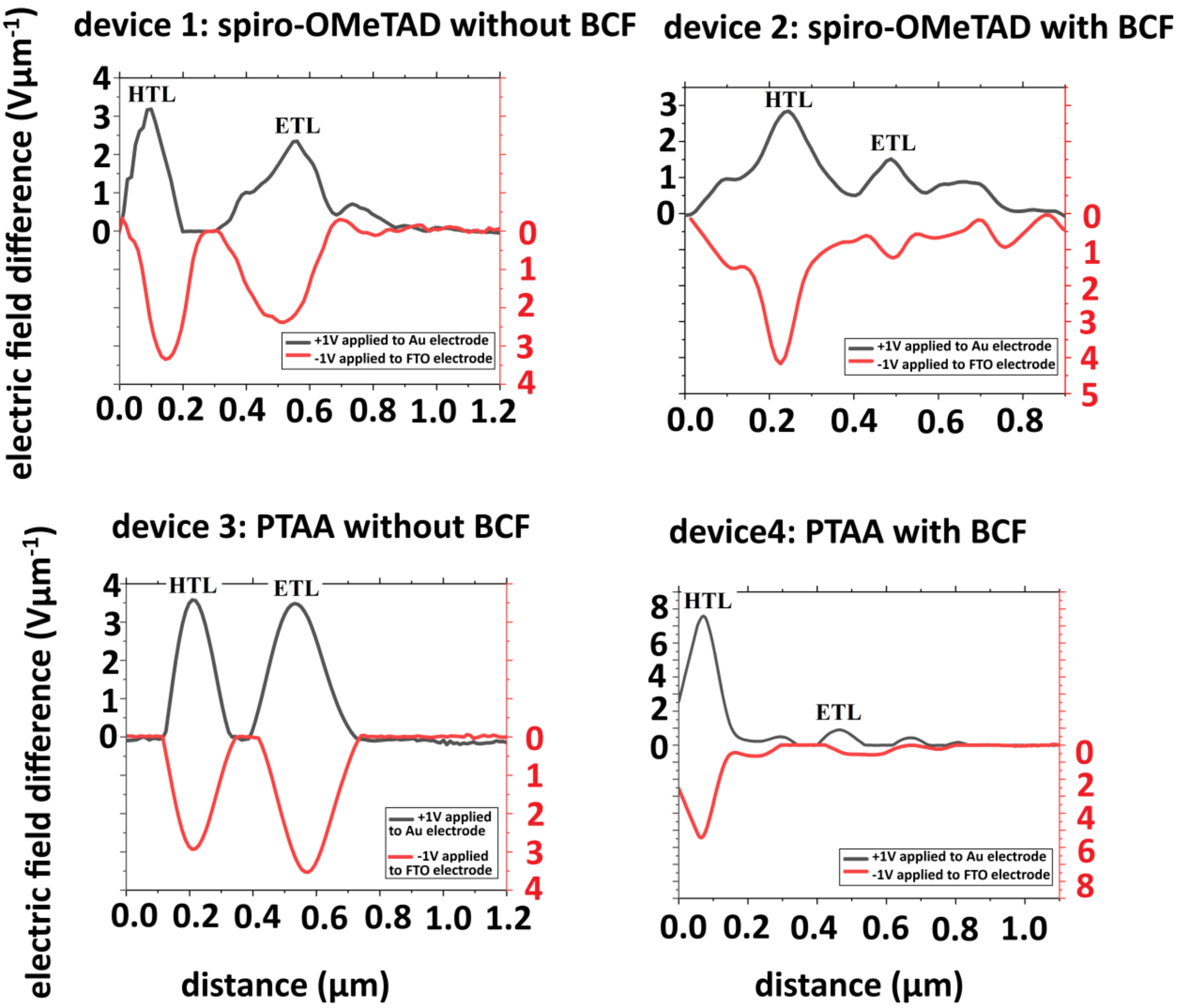

Figure S5: The results of the cross-sectional KPFM measurement with an applied voltage of +1 V on the Au side (FTO side grounded) and –1V on the FTO side (Au side grounded). We apply ±1 V because V_{oc} ≈ 1 V. Both configurations refer to a forward biased junction; therefore, their relative agreement is indicative of the reliability of our measurement. Both the black and the red vertical axes of the graphs correspond to positive values.

Section 6: Local variance of HTL and ETL layers

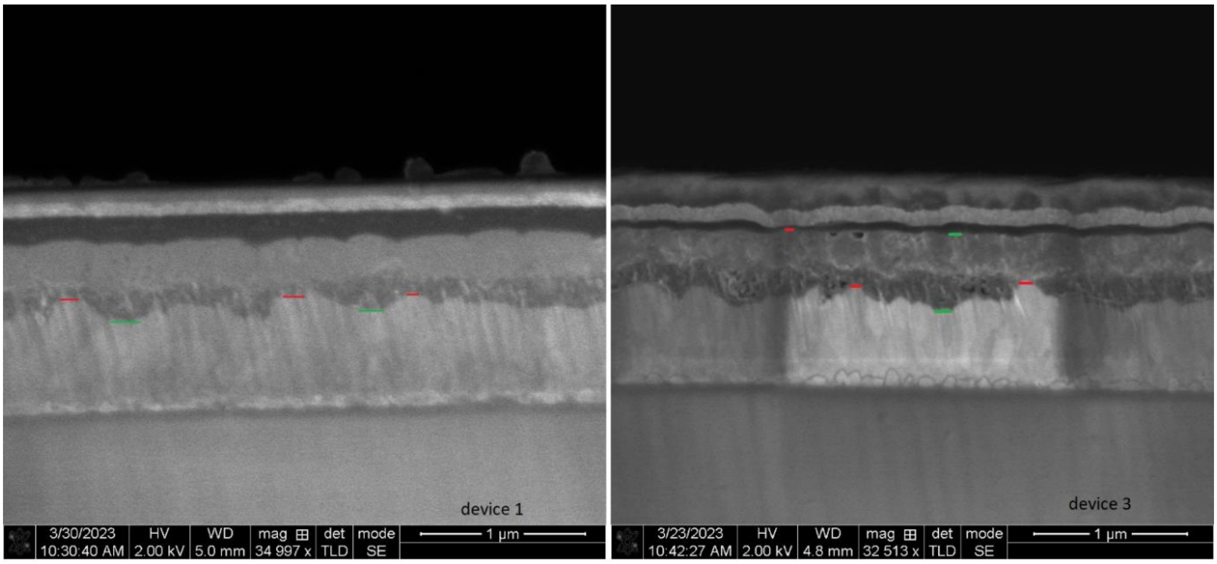

Figure S6: SEM pictures for devices 1 (spiro-OMeTAD) and 3 (PTAA), with indicators for the variation in thicknesses for the HTL and ETL layers.

As seen from the SEM images, there is a 150–200 nm discrepancy in the ETL layer width for all devices. For the PTAA devices, the maximum thickness difference for the HTL is 30 nm (red and green bars, distances measured with ImageJ). This variation can lead to discrepancies on the length of the voltage drop of up to 0.2 μm which affects the width of the electric field peaks.

Section 7: Analysis of peak heights and widths of the electric field difference graph via integration

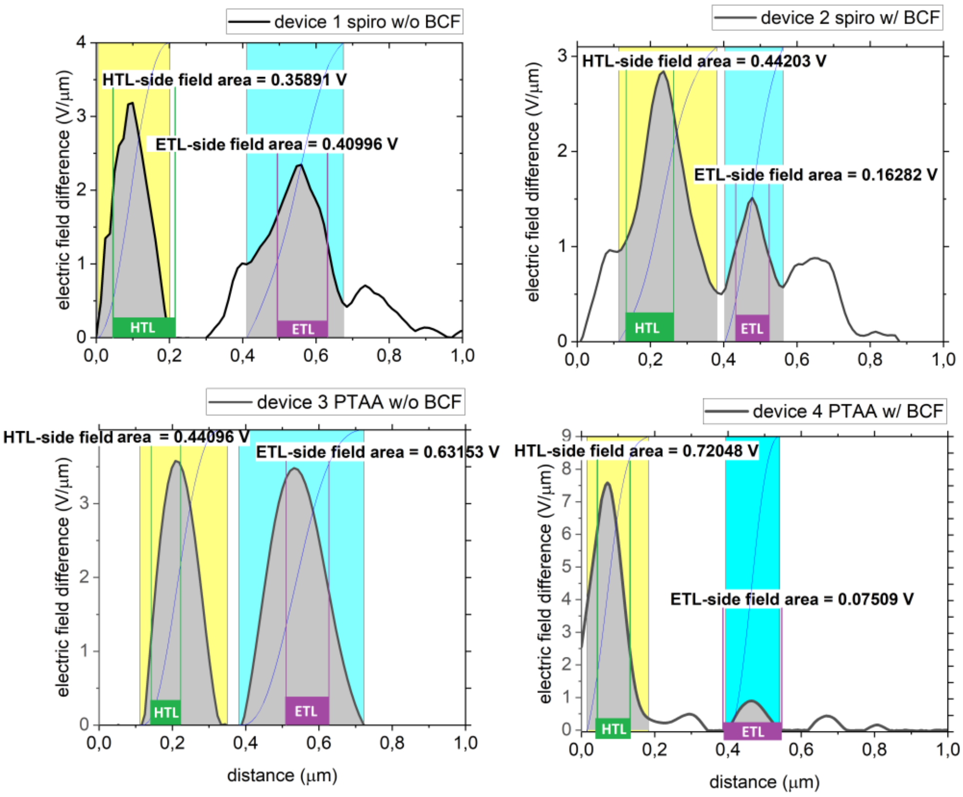

Figure S7: The integration of the HTL and ETL peaks is shown, which takes into consideration the peak heights and peak widths, since the voltage drop depends on both. The areas under the two peaks are unequal —ETL-side area is bigger— in devices 1 and 3, whereas the HTL peak shows a larger area than that of ETL for devices 2 and 4. This is due to the larger voltage drop there, since the HTL junction improved when BCF was added. The areas under the peaks that were considered for the integration were chosen so as to include the full electric fields at the junctions of the devices, which normally start from inside the perovskite absorber and expand through the transport layers. The result of the integration is a voltage value that is mathematically equal to the voltage drop magnitude at the perovskite/transport layer interface. The HTL and ETL layer widths are shown for reference, as they were identified from the SEM/AFM images, same as in Figure 3.

Section 8: Example KPFM images and potential profiles under illumination and open-circuit

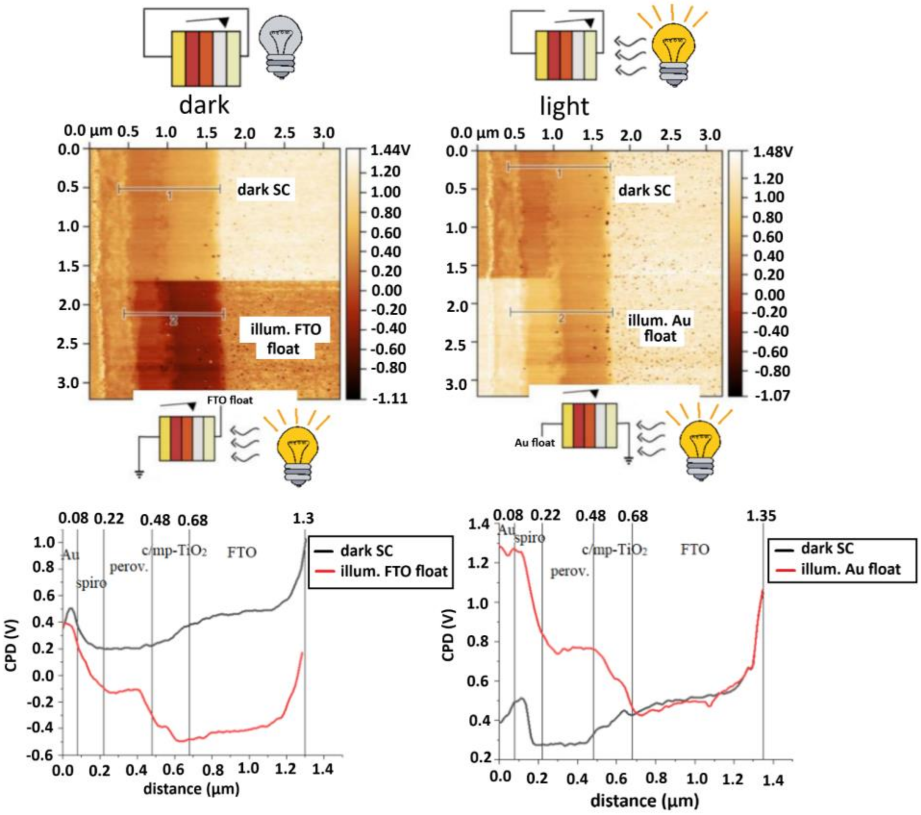

Figure S8: The dark measurement under short circuit reflects the relative work functions of the materials in contact. By subtracting it from consecutive open-circuit measurements, we get potential profiles that are only affected by the photogenerated charges. For the experiment under illumination, we sequentially float both the electrodes (FTO and Au) on two different measurements. We notice that when we float the FTO electrode under illumination, the potential drops by ≈V_{oc}, whereas when floating the Au electrode, the potential rises by ≈V_{oc}. This reflects the accumulation of electrons and holes to their respective contacts of the device. We can achieve the same configuration for the cell by applying a voltage ≈V_{oc} to the Au electrode, or ≈$-V_{oc}$ to the FTO electrode.

Section 9: KPFM image of the localized voltage drop at the HTL/perovskite interface (PTAA/BCF device)

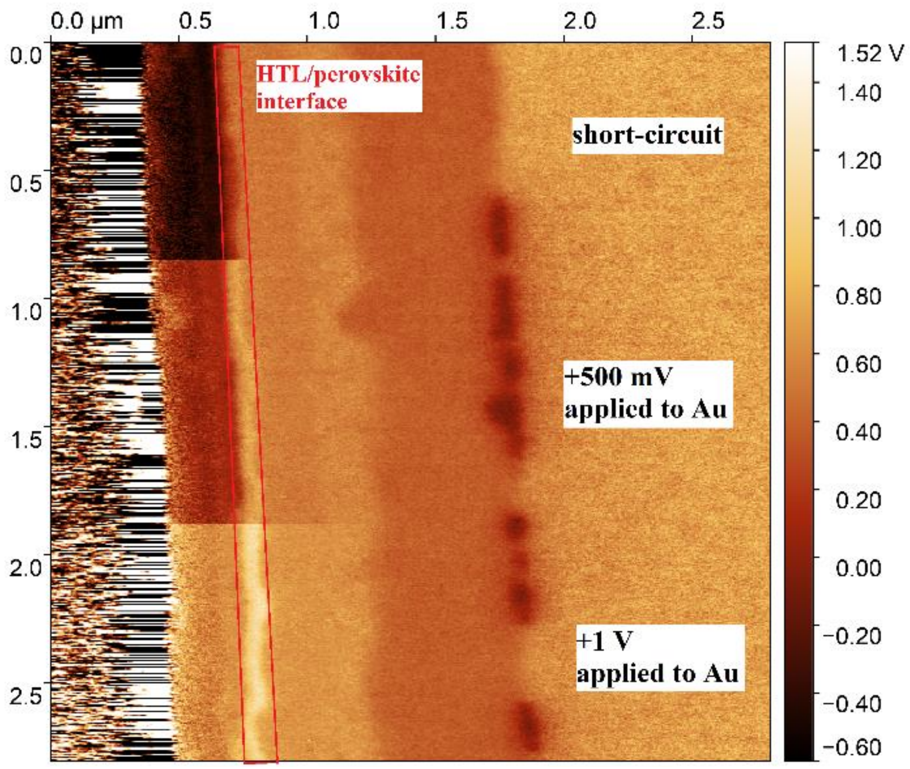

Figure S9: KPFM image of the PTAA/BCF cell, when different voltages are applied to the Au side. We notice how strong and localized the voltage drop is at the HTL interface, indicative of the improvement of the junction quality when BCF is incorporated into the PTAA precursor solution. From the left part of the image, we can see that as we scan towards the left edge, the signal becomes increasingly distorted, which is something that affects our local interpretation of the KPFM results near the Au side. That complication is referenced later in Supporting File 11.

Section 10: Comparison of photopotential profiles in illuminated devices when Au or FTO electrode remains floating

Looking at our graphs below, depicting the photovoltage curves (which are the "illuminated CPD - dark CPD" curves, Figure S8) we see p-i-n junctions where the total voltage drop should be the total amount of photovoltage (here approximately 1V, as is the V_{oc}). So the voltage from the high voltage part (Au) until the low voltage part (FTO) should drop in total by 1V, as shown in Figure S10. The voltage drops mainly at the diode junctions of the cells. If the two junctions (at perovskite /HTL and perovskite/ETL) are equally potent at extracting photocharges, then we expect the two junctions to exhibit a similar magnitude in voltage drop. This is the case with non-BCF devices 1 and 3. In the devices with BCF (2 and 4), which we proposed to have a better perovskite/HTL junction at extracting charges efficiently, there would need to be a larger drop at the HTL junction compared to that of the junction at the ETL side, since the overall voltage would still need to drop in the whole device by a total amount of 1V and the ETLs remained unaltered. The relative magnitude of the voltage drops at the two junctions reflects the competition of the two junctions to extract charges, with the better junction dropping the bigger share of the total of 1V which needs to drop in the whole device. This behaviour is caused by photo-charge built-up at the interfaces.

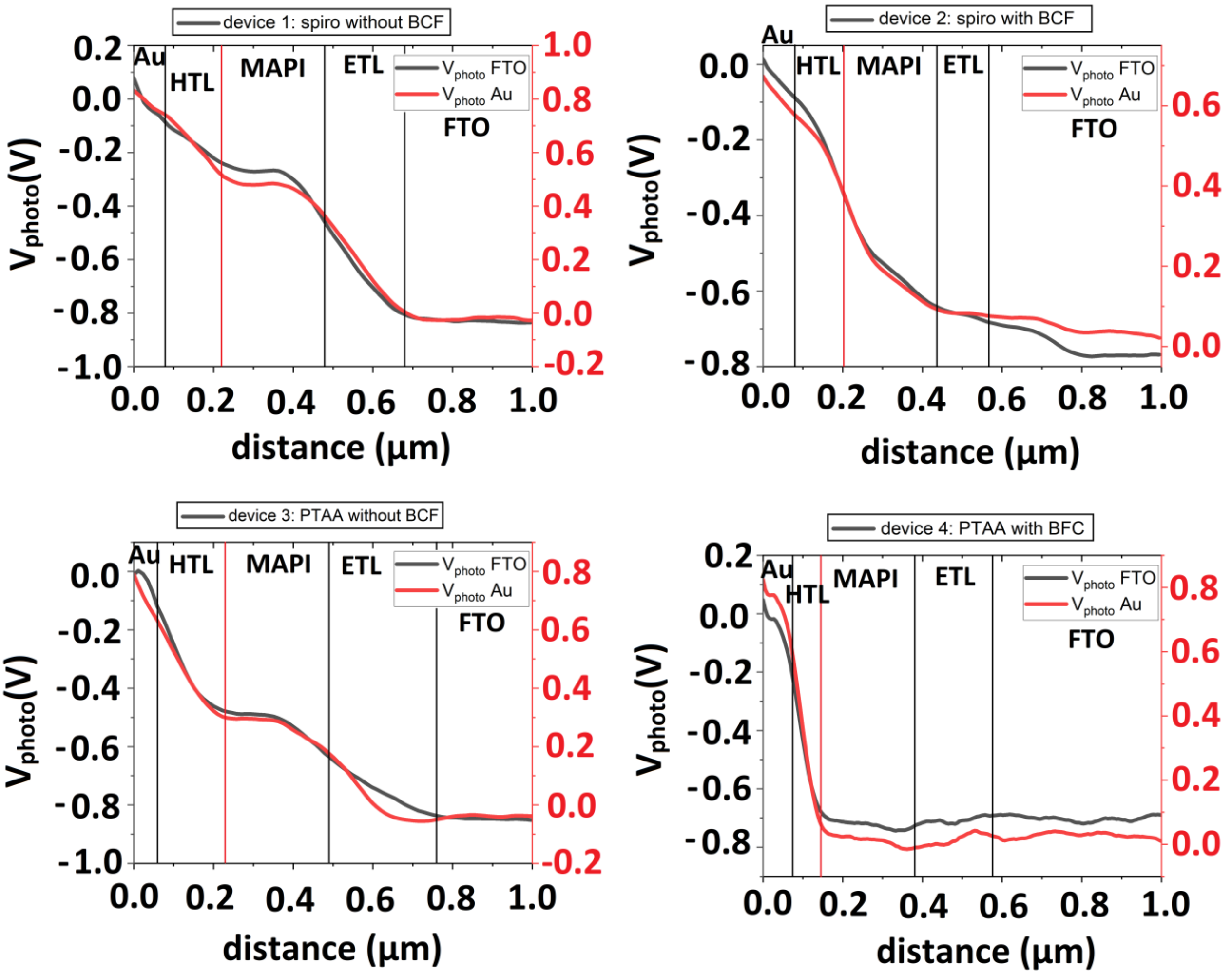

Figure S10: Photopotential profiles (CPD difference of illuminated profile minus dark/short-circuit profile) are plotted for the cases where FTO and Au electrodes are floating. The two distributions nearly coincide in every case, which indicates the high resolution of FM-KPFM and the elimination of crosstalk from the measurement [13].

Section 11: Complete measurement of cross-sectional KPFM under illumination and open circuit

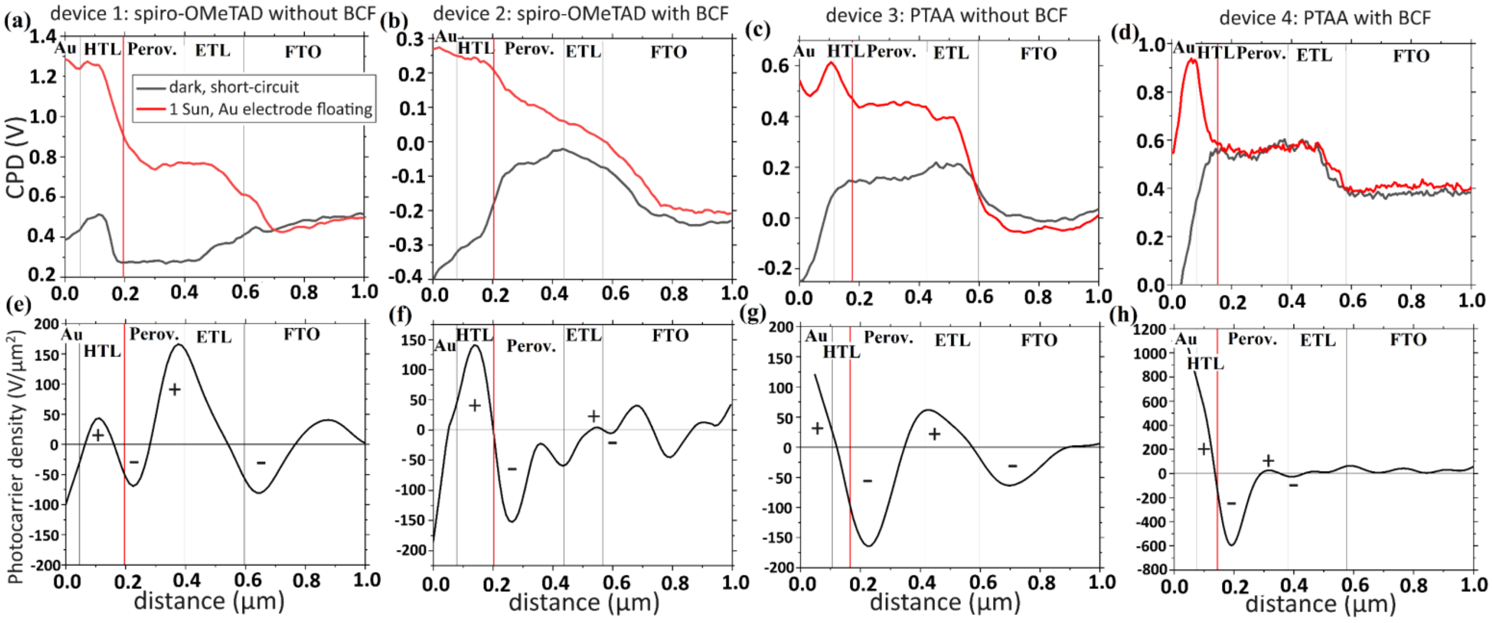

Figure S11: (a–d) The results of the cross-sectional KPFM measurement under illumination and open-circuit. The illumination was provided by a white-light source with irradiance similar to 1 Sun conditions. (e–h) Plots of the photo-charge density profiles ($\rho_{photo}(x)/\varepsilon_0\varepsilon$) across the device layers, with notations for positive and negative charge accumulation under open-circuit conditions.

We noted that the photovoltage profiles used for deriving the photocarrier density plots (Figure S11) were subjected to a 30 point adjacent-averaging method before any derivations. Without this step, the noise in the signal is amplified with each derivation step, leading to very noisy plots. Nevertheless, these signal processing steps make the signals less quantitative in terms of the exact magnitude and shape of the charge density profiles. This is particularly true for the signals close to the Au electrode, as here the tip scans towards the verge of the solar cell on each scan line (i.e., towards the Au side), where the measurement becomes noisier and more distorted since the tip approaches and falls off the steep edge after scanning a very thin layer of gold.

Nevertheless, the photo-charge densities, even at the Au side, can give a rough estimate of the position of the junction be means of the zero-crossing of the signals.

References for Supporting Information

1. Stephan, D. W. *Journal of the American Chemical Society* **2015**, 137, 10018-10032.

2. Stephan, D. W., Erker, G. *Angewandte Chemie International Edition* **2015**, 54, 6400-6441.

3. Peterson, K. A., Chabinyc, M. L. *Journal of Materials Chemistry C* **2022**, 10, 6287-6295.

4. Luo, J., Xia, J., Yang, H., Chen, L., Wan, Z., Han, F., Jia, C. *Energy & Environmental Science* **2018**, 11, 2035-2045.

5. Ye, T., Chen, W., Jin, S., Hao, S., Zhang, X., Liu, H., He, D. *ACS applied materials & interfaces* **2019**, 11, 14004-14010.

6. Koh, C. W., Heo, J. H., Uddin, M. A., Kwon, Y. W., Choi, D. H., Im, S. H., Woo, H. Y. *ACS applied materials & interfaces* **2017**, 9, 43846-43854.

7. Khadka, D. B., Shirai, Y., Yanagida, M., Ryan, J. W., Miyano, K. *Journal of Materials Chemistry C* **2017**, 5, 8819-8827.

8. Li, Z., Tinkham, J., Schulz, P., Yang, M., Kim, D. H., Berry, J., Zhu, K. *Advanced Energy Materials* **2016**, 7(NREL/JA-5900-66721).

9. Ye, T., Wang, J., Chen, W., Yang, Y., He, D. *ACS Applied Materials & Interfaces* **2017**, 9, 17923-17931.

10. Ren, G., Han, W., Deng, Y., Wu, W., Li, Z., Guo, J. Guo, W. *Journal of Materials Chemistry A* **2021**, 9, 4589-4625.

11. Abate, A., Leijtens, T., Pathak, S., Teuscher, J., Avolio, R., Errico, M. E., Snaith, H. J. *Physical Chemistry Chemical Physics* **2013**, 15, 2572-2579.

12. Liu, J., Liu, W., Aydin, E., Harrison, G. T., Isikgor, F. H., Yang, X., De Wolf, S. *ACS applied materials & interfaces* **2020**, 12, 23874-23884.

13. Bergmann, V. W., Guo, Y., Tanaka, H., Hermes, I. M., Li, D., Klasen, A., Weber, S. A. *ACS applied materials & interfaces* **2016**, 8, 19402-19409.
